# A Case of Severe Ulcerative Colitis with Colonic Dilatation caused by Renal Mucinous Tubular and Spindle Cell Carcinoma

**DOI:** 10.5005/jp-journals-10018-1198

**Published:** 2016-12-01

**Authors:** Monika Kukulska, Izabela Smola, Agnieszka Halon, Leszek Paradowski, Elzbieta Poniewierka, Radoslaw Kempinski, Abdulhabib Annabhani

**Affiliations:** 1 Department of Gastroenterology and Hepatology, Wroclaw Medical University, Wroclaw, Polland; 2 Department of Pathomorphology and Oncological Cytology, Wroclaw Medical University, Wroclaw, Polland

**Keywords:** Mucinous tubular and spindle cell renal cell carcinoma, Toxic megacolon, Ulcerative colitis.

## Abstract

We present a case of ulcerative colitis (UC) in a patient during the first severe relapse with colonic dilatation and coexisting of giant renal tumor. Kidney tumor was constricting on colon and imitating toxic megacolon (TM). The patient with severe acute colitis (SAC) was admitted to the Department of Gastroenterology and Hepatology with inflammatory bowel disease (IBD) diagnosed in the previous month, based on clinical symptoms, endoscopy, and histopathology results. Computed tomography (CT) revealed a giant left kidney tumor without evidence of infiltration, a dilatation of the transverse colon up to 6 cm, and narrow light of the descending colon. There were no signs of intestinal obstruction or perforation. The control X-rays revealed maintaining megacolon, with dilatation of splenic flexure projection up to 6.5 to 7.5 cm. The patient was treated conservatively with no apparent improvement and finally operated on. Intraoperatively, a large tumor of the kidney (12 cm) constricting intestine was revealed. Left-sided nephrectomy and partial resection of the colon with the emergence of a colostomy was performed. The histopathology exam revealed renal mucinous tubular and spindle cell carcinoma (RMTSCC), a very rare malignant kidney tumor of low malignant potential and relative good prognosis. It was identified in the past 20 years. To date, approximately 100 such cases of cancer have been described.

**How to cite this article:**

Kukulska M, Smola I, Halon A, Paradowski L, Poniewierka E, Kempinski R, Annabhani A. A Case of Severe Ulcerative Colitis with Colonic Dilatation caused by Renal Mucinous Tubular and Spindle Cell Carcinoma. Euroasian J Hepato-Gastroenterol 2016;6(2):190-193.

## INTRODUCTION

Ulcerative colitis (UC) is a chronic and diffuse inflammation of the colonic mucosa. The disease proceeds with periods of exacerbation and remission. Severe acute colitis (SAC) occurs in 12 to 25% of cases. All patients with SAC should be hospitalized. According to the European Crohn’s and Colitis Organization (ECCO) criteria, SAC is defined as the presence of six or more loose stools with blood per day (mandatory criterion), tachycardia (>90/min), elevated temperature (>37.8° C), anemia (hemoglobin concentration <10.5 gm/dL), or increased erythrocyte sedimentation rate (ESR) >30 mm/hour. The treatment of choice is steroids administered intravenously. Endoscopic procedures should be avoided, and laxative-based bowel preparation formulas should be used with caution as they are risk factors for the development of toxic megacolon (TM).^1^ Complications of the disease like TM and colon perforation are burdened with a very high mortality rate. Prolonged conservative treatment increases the risk of complications. Sometimes urgency surgical treatment is needed as a life-saving procedure.^1^

We present a case of patient with SAC with symptoms of TM confirmed in the computed tomography (CT) and several X-rays. The diagnosis process also revealed left kidney tumor constricting the large intestine and causing distension of the proximal colon, as determined during surgery. Nephrectomy and partial resection of descending colon and sigmoid junction, with emergence of colostomy, was finally performed. The histopathology exam revealed renal mucinous tubular and spindle cell carcinoma (RMTSCC) which stand for a very rare malignant kidney tumor, identified in the past 20 years, of low malignant potential and relative good prognosis.^2^

## CASE REPORT

A 53-year-old male with UC and coexisting renal tumor, after embolization of left renal artery and with planned nephrectomy, was admitted to the Department of Gastroenterology and Hepatology in December of 2014 due to abdominal pain, fever, tachycardia, and severe diarrhea with bloody stools.

Ulcerative colitis was diagnosed in November 2014, during hospitalization in the Department of Surgery, where the patient was treated for permeable intestinal obstruction. In the performed incomplete colonoscopy, coaxial inflammatory infiltration with accompanying diverticulum was visualized in the descending colon. Moreover, in the descending colon, sigmoid colon and rectum advanced lesions corresponding with UC were described. Histopathological examination confirmed the diagnosis.

Blood test performed on admission revealed iron-deficiency anemia with a hemoglobin level of 10.4 gm/dL, raised inflammatory markers (C reactive protein, ferritin, accelerated ESR), and low cholesterol. Due to decreased albumin, the patient received human albumin solution. Stool tests ruled out *Clostridium difficile, Salmonella, Shigella, Yersina, Campylobacter*, and enterohemorrhagic *Escherichia coli* (EHEC) infection.

**Figs 1A to C:**
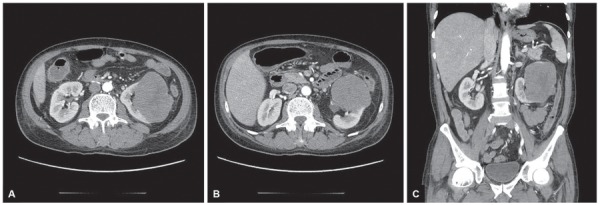
Computed tomography scans revealed a giant left kidney tumor

Computed tomography, except with active inflammatory lesions extending from transverse colon to rectum, revealed dilatation of transverse colon up to 6 cm. There were no signs of intestinal obstruction or perforation. Within the lower pole of the left kidney, CT revealed the huge, mostly heterogeneous, well-bounded tumor size 7 × 9.5 × 10 cm (Figs 1A to C). There was no evidence of tumor infiltration beyond the kidney. Visible lymph nodes were enlarged to 1.3 × 0.9 cm along the left renal vessels.

The patient was treated with intravenous antibio­tics (ciprofloxacin in combination with metronidazole), hydrocortisone, and fluids. He also received anticoagulant and masalazine. Despite of intensive treatment and parenteral nutrition, abdominal X-rays showed no sign of improvement. Progression in the dilatation of transverse colon up to 7.5 cm was visualized. We decided to transfer the patient to the Department of Surgery, where he was treated surgically. Intraoperatively, kidney tumor was found as the cause of the obstruction and dilatation of the colon. Nephrectomy and partial resection of descending colon sigmoid junction, with emergence of colostomy, was performed.

**Figs 2A to F:**
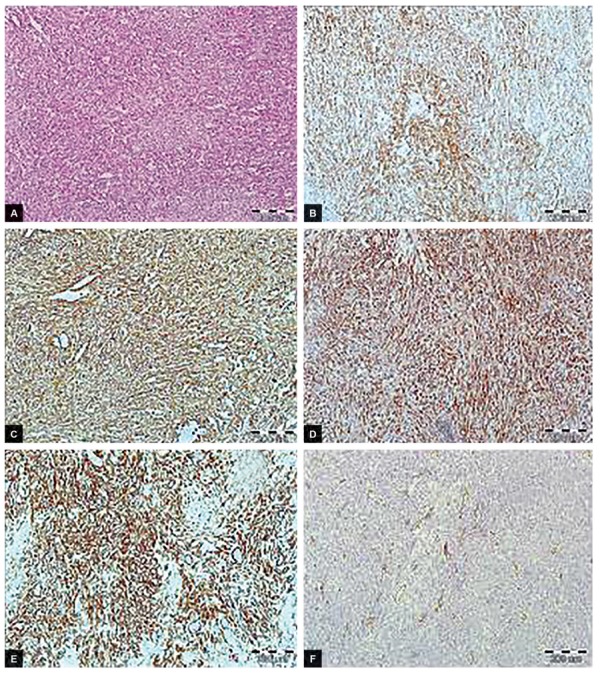
Mucinous tubular and spindle cell renal carcinoma. Tumor cells with relatively low-grade cytology are forming collapsed tubules, resulting in a spindle-shaped appearance (Fig. 2A, HE, 100x). Typical immunohistochemical profile of tumor with positive expression of vimentin (Fig. 2B, 100x), CK7 (Fig. 2C, 100x), Alpha-methylacyl-CoA racemase (AMACR) (Fig. 2D, 100x), renal cell carcinoma (RCC) (Fig. 2E, 100x) and negative reaction with CD10 (Fig. 2F, 100x)

The histopathology exam revealed RMTSCC with low malignancy potential, showing no mitotic activity, and with size of 12 cm diameter (Figs 2A to F). Neoplasm without fatty tissues infiltration and structures of renal hilus or renous vessels, limited to renal parenchyma, was found.

In March 2015, the patient was admitted to the Department of Gastroenterology and Hepatology again due to fever, anemia and elevated inflammation markers. The CT images have raised suspicion of abscess in tumor bed and between bowel loops. The patient was treated surgically once again. During surgery, the abscess, however, was not confirmed. Still there was a progression of inflammation of the colon; so colectomy, appendectomy, and partial resection of ileum were performed.

## DISCUSSION

Toxic megacolon is total or segmental nonobstructive colonic dilatation of at least 6 cm. It is a rare but serious complication that occurs among 1.6 to 3% of UC patients, usually during the first severe relapse with involvement of the left half of the colon or pancolitis.^3^ Forty percent of patients with TM or fulminant colitis require urgent surgery.^4^ Toxic megacolon can be also a complication of any kind of intestinal infections (*Salmonella, Shigella, Campylobacter, Cytomegalovirus*), ischemic colitis, radiation, or obstructive colorectal cancer.^5,6^ The pathophysiology of toxic colonic dilatation is poorly known.^5^ It is possible that myenteric plexi is involved in its development, causing paralytic dilation of the bowel.^4^

After 48 hours of ineffective conservative treatment of MT, an urgent colectomy should be considered. In the case of urgent indications, and in patients with serious condition overall, procedure should be performed in two stages. Subtotal colectomy with preservation of the rectum and temporary ileostomy is a standard practice. In the second stage, restoration of intestinal continuity with ileal pouch-anal anastomosis (IPAA) should be considered.^4^

In our case report, SAC with coexisting big kidney tumor (12 cm diameter) was shown. They were accidentally identified at the same time during hospitalization of the patient in the Department of Surgery. It did not seem that renal tumor in any way influence the UC and induce complications. In CT scans and ultrasound of the abdomen, there were no signs of constricting the intestinal. Tumor was well demarcated without evidence of infiltration beyond the kidney. Therefore, the patient had embolization of tumor artery firstly and qualified for surgery to remove a kidney tumor urgently after control of exacerbations of inflammatory bowel disease (IBD).

The patient was finally operated due to progression in the dilatation of transverse colon. Left-sided nephrectomy and only partial resection of the colon with the emergence of a colostomy was performed. It was not necessary to perform colectomy, which was favorable for the patient.

The histopathology exam revealed RMTSCC. Renal mucinous tubular and spindle cell carcinoma is a rare type of renal cell carcinoma. It has been described as a distinct entity in the 2004 World Health Organization (WHO) tumor classification.^7^ To date, approximately 100 cases of cancer have been described. It occurs four times more in women than in men, in a wide range of age between 20 and 81 years.^8^ The size of the tumors recently reported varies between 1 and 18 cm.^2^ An association with nephrolithiasis has been noted.^9^ The prognosis for RMTSCC with classic morphology is usually good. Surgical excision (partial or radical nephrectomy) in most cases is a sufficient treatment; however, a close follow-up should be considered.^7^ Actually, to our knowledge, there are no reports of coexistence RMTSCC with IBD.

## SUMMARY

Our case shows that megacolon was not a direct complication of the disease, but mechanical compression of the intestine by a kidney tumor. This shows the importance of difficulty in differential diagnosis and management. In conclusion, it is important to consider that the colonic dilatation can be caused by oppressive from the outside, even in a patient with SAC. In such a patient, high-risk emergency colectomy can be avoided. Therefore, the implementation of CT in patients with an unusual course of the disease is worth considering.
